# Local Irradiation Modulates the Pharmacokinetics of Metabolites in 5-Fluorouracil—Radiotherapy–Pharmacokinetics Phenomenon

**DOI:** 10.3389/fphar.2020.00141

**Published:** 2020-02-27

**Authors:** Ju-Han Liu, Tung-Hu Tsai, Yu-Jen Chen, Li-Ying Wang, Hsin-Yu Liu, Chen-Hsi Hsieh

**Affiliations:** ^1^ School of Nursing, National Taipei University of Nursing and Health Sciences, Taipei, Taiwan; ^2^ Institute of Traditional Medicine, National Yang-Ming University, Taipei, Taiwan; ^3^ Graduate Institute of Acupuncture Science, China Medical University, Taichung, Taiwan; ^4^ School of Pharmacy, College of Pharmacy, Kaohsiung Medical University, Kaohsiung, Taiwan; ^5^ Department of Chemical Engineering, National United University, Miaoli, Taiwan; ^6^ Departments of Radiation Oncology, Mackay Memorial Hospital, Taipei, Taiwan; ^7^ Department of Medical Research, China Medical University Hospital, Taichung, Taiwan; ^8^ School and Graduate Institute of Physical Therapy, College of Medicine, National Taiwan University, Taipei, Taiwan; ^9^ Physical Therapy Center, National Taiwan University Hospital, Taipei, Taiwan; ^10^ Department of Family Medicine, Taipei Veterans General Hospital, Taipei, Taiwan; ^11^ Division of Radiation Oncology, Department of Radiology, Far Eastern Memorial Hospital, New Taipei City, Taiwan; ^12^ Faculty of Medicine, School of Medicine, National Yang-Ming University, Taipei, Taiwan

**Keywords:** 5-fluorouracil, 5-fluoro-5,6-dihydrouracil, radiotherapy, pharmacokinetics, HPLC-UV, irradiation

## Abstract

**Background:**

The effects of radiotherapy (RT) on the pharmacokinetics (PK) of 5-FU and 5-fluoro-5,6-dihydro-uracil (5-FDHU) were investigated by animal experiments.

**Methods:**

Whole-pelvis RT with 0.5 and 2 Gy was delivered to Sprague–Dawley rats. 5-FU at 100 mg/kg was intravenously infused 24 h after radiation. The pharmacokinetics of 5-FU and 5-FDHU in the plasma and bile system were calculated.

**Results:**

The areas under the concentration versus time curve (AUC) of 5-FU in the plasma were reduced by local irradiation by 23.7% at 0.5 Gy (P < 0.001) and 35.3% at 2 Gy (P < 0.001). The AUCs of 5-FDHU were also reduced by 21.4% at 0.5 Gy (P < 0.001) and 51.5% at 2 Gy (P < 0.001). Irradiation significantly increased the clearance values (CLs) of 5-FU by 30.6% at 0.5 Gy and 50.1% at 2 Gy, respectively. The CLs of 5-FDHU were increased by 27.2% at 0.5 Gy and 106% at 2 Gy. The AUCs of 5-FU in the bile were increased by 36.7% at 0.5 Gy (P < 0.001) and 68.6% at 2 Gy (P = 0.005). The AUCs of 5-FDHU in the bile were increased by 40.3% at 0.5 Gy (P < 0.001) and 248.1% at 2 Gy (P < 0.001). The CLs of 5-FU in the bile were increased by 31.8% at 0.5 Gy and 11.2% at 2 Gy. However, the CLs of 5-FDHU in the bile were decreased by 29.1% at 0.5 Gy and 71.0% at 2 Gy.

**Conclusion:**

Both conventional and low-dose irradiation can affect the pharmacokinetics of 5-FU and its metabolite, 5-FDHU. RT plus 5-FU could cause more adverse events than 5-FU alone by increasing the AUC ratio of 5-FU/5-FDHU. Irradiation decreases the AUC of 5-FU in the plasma, which may cause poor clinical outcomes.

## Introduction

Five-fluorouracil (5-FU) a traditional chemotherapeutic agent used in concurrent chemoradiation therapy (CCRT) to enhance the radiotherapy (RT) effects in rectal cancer patients (Fisher et al., 1988). Compared to surgery or RT alone, adjuvant CCRT (Wolmark et al., 2000) or neoadjuvant CCRT (Bosset et al., 2006) improves the locoregional control and overall survival in rectal cancer patients by 10%–15%.

More than half of the catabolic activity of 5-FU in the liver through the dihydropyrimidine dehydrogenase (DPD) pathway generates toxic 5-fluoro-5,6-dihydro-uracil (5-FDHU), followed by fluoroureidopropionic acid and α-fluoro-β-alanine (Wasternack, 1980; Heggie et al., 1987; Bocci et al., 2000). The cytosolic enzyme DPD in the catabolism of 5-FU is widely expressed in the body (Lu et al., 1993; Van Kuilenburg et al., 1997; Mcleod et al., 1998). The increased AUC ratio of 5-FU/5-FDHU is associated with adverse events (Di Paolo et al., 2001), and the modulation of the catabolic pathway of 5-FU has an impact on the side effects and adverse reactions (Ezzeldin and Diasio, 2004).

Pharmacokinetics is the study of the kinetics of a drug and/or its metabolites in the body and what the body does to the drugs (1991). In the past, RT has been used as a local treatment (Elsasser and Scholz, 2007). Growing evidence shows that the systemic pharmacokinetics (PK) of anticancer drugs can be modulated by local RT with different RT doses; this is called the RT-PK phenomenon (Hsieh et al., 2010; Hsieh et al., 2013; Hsieh et al., 2015; Chen et al., 2017). The area under the plasma concentration *versus* time curve (AUC) of 5-FU is reduced by RT (Hsieh et al., 2015), and the excretion of 5-FU is facilitated by RT (Hsieh et al., 2011).

However, whether RT modulates the PK of the metabolite of 5-FU, 5-FDHU, remains unclear. The current study investigates the interaction between RT and the pharmacokinetics of 5-FU and its metabolite in rats. The goal of the current study is to provide clinicians with more information about the interaction between RT, 5-FU, and 5-FDHU and to improve daily practice.

## Materials and Methods

### Reagents and Materials

5-FU, 5-FDHU, amoxicillin (internal standard), and urethane were provided by Sigma-Aldrich Chemicals (St. Louis, MO, USA). Potassium dihydrogen phosphate (KH_2_PO_4_), potassium hydroxide (KOH), phosphoric acid (H_3_PO_4_), and HPLC-grade methanol were purchased from E. Merck (Darmstadt, Germany). For all aqueous solutions in the experiment, deionized water from Millipore (Milford, MA, USA) was used.

5-FU and 5-FDHU were dissolved in methanol to produce a standard solution (1 mg/ml) and were diluted into Eppendorf tubes as a stock solution (10 μg/ml). The working solution was prepared by diluting the stock solution in 50% (v/v) methanol to obtain the following concentrations: 0.1, 0.5, 1, 5, 10, and 50 μg/ml. All stock solutions were stored in darkness at −20°C.

### Instrumentations and HPLC-UV Conditions

The HPLC system consisted of chromatographic pumps (LC-20AT; Shimadzu Co., Kyoto, Japan), an autosampler (SIL-20AC; Shimadzu Co., Kyoto, Japan), and a UV-Vis detector (SPDM20A; Shimadzu Co., Kyoto, Japan). All analytical samples were separated using a reverse-phase Diamonsil C18 column (250 mm × 4.6 mm i.d.; particle size 5 μm, Dikma, Lake Forest, China). The mobile phase for HPLC analysis consists of two solvent compositions: 10 mM potassium dihydrogen phosphate (KH_2_PO_4_) and methanol (95: 5, v/v). The pH of 10 mM KH_2_PO_4_ was adjusted to pH 4.7 using phosphoric acid or potassium hydroxide. The flow rate for the mobile phase was set at 1 ml/min. The temperature in the autosampler was set at 4°C, the analytical volume was 10 μl for each sample, the UV-Vis detector scanned from 200 to 500 nm, and the chromatographic profiles were monitored at 215 nm for 5-FU and 5-FDHU.

### Preparation of 5-FU and 5-FDHU Plasma Extraction

The sample extract preparation was conducted as follows. First, 50 μl of rat plasma was mixed with 10 μl of internal standard (amoxicillin) solution and 140 μl of methanol for protein precipitation. The samples were vortex-mixed for 5 min and centrifuged at 13,000 × g at 4°C for 10 min. The supernatants were purified through a 0.22-μm supernatant filter prior to HPLC–UV analysis.

## Method Validation

### Calibration Curves

The calibration curves ranged in concentration from 0.1 to 50 μg/ml for the blood. The linearity of the assay was checked using the coefficient of determination (*r^2^*) for the calibration curve, which should be greater than 0.995. The limit of detection (LOD) was determined at the concentration that generates a signal-to-noise ratio of 3, and the lower limit of quantification (LLOQ) was defined as the lowest concentration of the linear regression that yields a signal-to-noise ratio of 10.

### Extraction Recovery

5-FU and 5-FDHU were diluted to 0.5, 5, and 50 μg/ml in the mobile phase. Set 1: The stock solutions of 5-FU and 5-FDHU were mixed with 10 μl of amoxicillin (I.S.) solution and diluted to 0.5, 5, and 50 μg/ml in the mobile phase. Set 2: A total of 10 μl of standard solution was added to 50 μl of blank plasma, 10 μl of amoxicillin (I.S.) solution, and 130 μl of methanol and prepared as described in the sample preparation section. A pre-extraction sample of 5-FU and 5-FDHU was prepared and used for HPLC–UV analysis. The recovery was calculated as the peak area of Set 2 divided by the peak area of Set 1.

### Accuracy and Precision Evaluation

The accuracy and precision evaluation methods were based on the Food and Drug Administration (FDA) guidelines [34]. The accuracy was estimated as bias (%) = (observed concentration − nominal concentration) × 100/nominal concentration. The precision was calculated as the relative standard deviation, RSD % = (SD) × 100/observed concentration. The inter- and intra-day precision (% RSD) and accuracy (% bias) were less than 15% for all analytes at low, medium, and high QC concentrations according to the biological method validation guidelines of the FDA [34]. The precision and accuracy of this analytical method were verified by preparing six identical calibration curves on the same day (intraday) and on six successive days (interday). In addition, five conditions were evaluated for the stability study, including short-term storage at room temperature, long-term storage, three freeze-thaw cycles, post-preparative, and stock solution stability. Calibrations in six replications on the same day (intra-day) and on six successive days (inter-day) were achieved to verify the accuracy and precision. 5-FU and 5-FDHU were prepared at concentrations of 0.1, 0.5, 1, 5, 10, and 50 μg/ml. The calibration curve was described using the peak area ratio of 5-FU and 5-FDHU hydrochloride versus the concentration.

### Stability Evaluation

According to the FDA guidelines, the stabilities of 5-FU and 5-FDHU were evaluated using the following methods. 1) Short-term: The samples were stored at room temperature (25 ± 3°C) for 4 h before analysis. 2) Post-preparative: The samples were kept at 8°C for 8 h in an autosampler before analysis. 3) Freeze and thaw: The samples were stored at −20°C for 24 h and then thawed at room temperature. The freeze and thaw cycles were repeated three times. 4) Long-term: The samples were kept at −20°C for 30 days in darkness before analysis.

Concentrations of 0.5, 5 and 50 μg/ml of 5-FU and 5-FDHU were selected to measure stability. The relative error between the freshly prepared samples and the stored samples was calculated to determine the stability. The limitation of sample stability was defined as within ± 15%, and LLOQ values were less than ± 20%.

## Experimental Animals

The protocol was reviewed and approved by the Institutional Animal Experimentation Committee of National Yang-Ming University, Taipei, Taiwan, and by the Institutional Animal Care and Use Committee (IACUC, approval number 106DN22). Male Sprague-Dawley rats (250–280 g) were provided by the Laboratory Animal Center at National Yang-Ming University (Taipei, Taiwan). The animals had access to water ad libitum and food (laboratory rodent diet 5P14, PMI Feeds, Richmond, IN, USA) and lived in a pathogen-free environment with a 12-h light-dark cycle. All animal experiments followed the guidelines and procedures for the care of laboratory animals at National Yang-Ming University.

**Figure 1:**
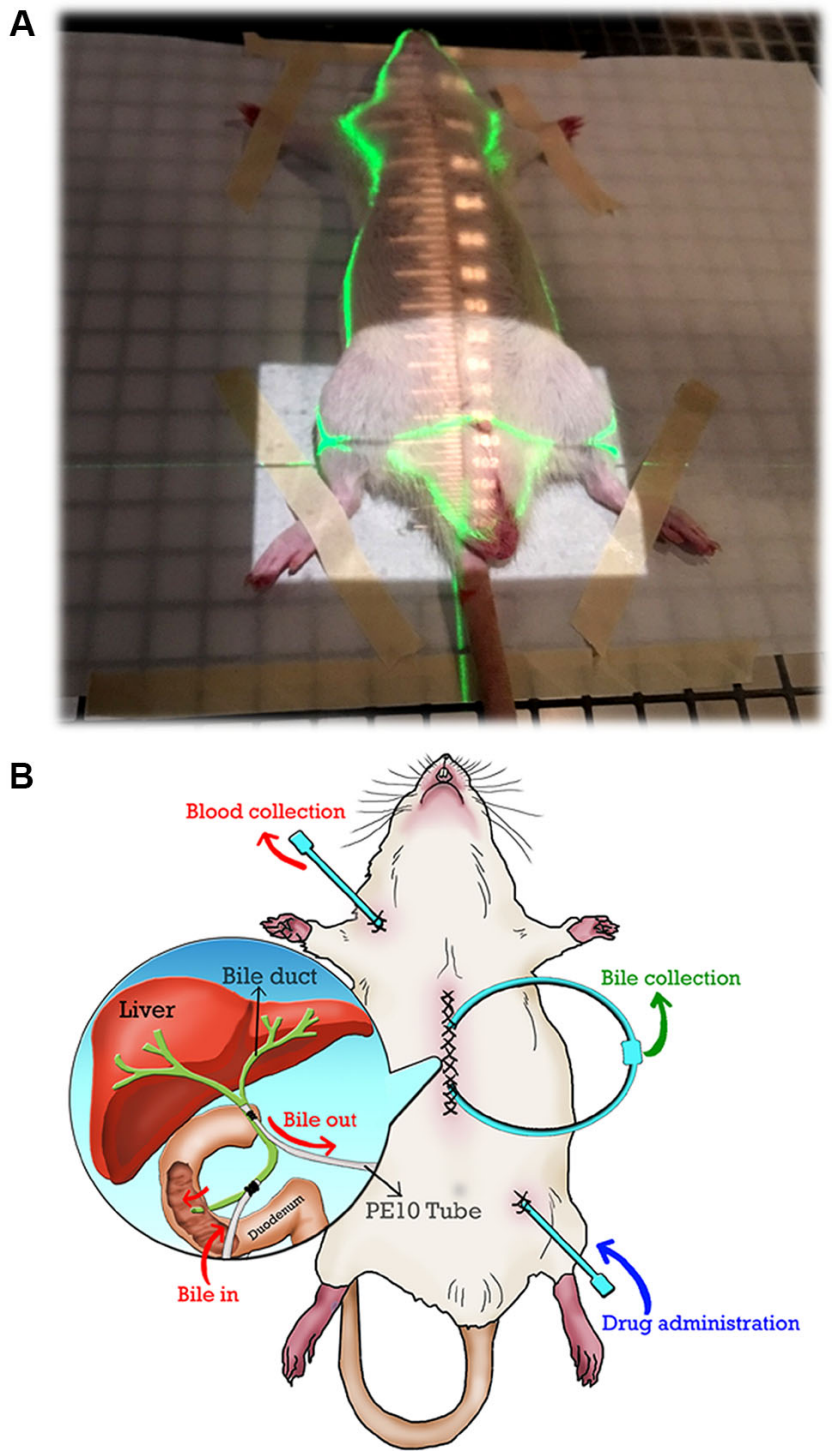
**(A)** The irradiation field of the whole pelvis. The cranial margin was set at the top of the bilateral iliac crest for the whole pelvic field. Conventional radiotherapy was used to deliver the radiation dose *via* the anterior-posterior (AP) and PA portals. **(B)** Illustration of the bile duct cannulated rat model under anesthesia in this study. Exteriorization of the PE10 tube was prepared before bile duct catheterization. PE10 cannula was fixed to the bile duct (two positions near the liver and duodenum) by a surgical knot, and the wound was closed by sutures after duct cannulation was completed. The bile of the rat can flow into the circulation system when the pipes of the bile ducts are connected. 5-FU was administered *via* PE50 cannula into the femoral vein, and blood was collected through PE50 cannula from the jugular vein.

The rats were anesthetized with urethane 1 g/ml and achloralose 0.1 g/ml (1 ml/kg by intraperitoneal injection) and were immobilized on a board when undergoing computed tomography for the imaging of the whole pelvic field. Conventional radiotherapy was used to deliver the radiation dose *via* the anterior-posterior (AP) and PA portals (**Figure 1A**) (Hsieh et al., 2011). The experimental animals were randomized to the control, 0.5 Gy followed by 5-FU and 2 Gy followed by 5-FU groups. Each group’s data were collected from six rats. Dosages of 0.5 and 2 Gy for the rats and 100 mg/kg as a feasible 5-FU dose in rats to examine the 5-FU pharmacokinetic parameters were determined from the results of previous reports (Hsieh et al., 2010; Hsieh et al., 2011; Hsieh et al., 2015). Twenty hours after RT, the rats were administered 100 mg/kg of 5-FU in 2 ml of normal saline by intravenous infusion into the femoral vein over a 2-min period (Hsieh et al., 2010) (**Figure 1B**). A 150-μl blood sample was withdrawn from the jugular vein with a fraction collector according to a programmed schedule at 5, 15, 30, 45, and 60 min and 1.5, 2, 2.5, and 3 h following drug administration. The total blood volume of rodents is approximately 7% of their body weight, and approximately 20% of total blood volume can be withdrawn (Lee and Goosens, 2015). Thus, the volume of a blood sample that can be collected from a Sprague-Dawley rat is approximately 19.6 ml. In the current study, the volume of blood sample for each time collection was 150 μl, so the total blood volume collection was below 10% of the total circulating blood volume to avoid affecting the physiological index of the rats. The blood samples were immediately centrifuged at 3300× *g* for 10 min. The resulting plasma (50 μl) was added to 1 ml of ethyl acetate in a clean tube, vortexed for 5 min, and centrifuged at 5900 × *g* for 10 min. After centrifugation, the upper organic layer containing the ethyl acetate was transferred to a new tube and evaporated to dryness under flowing nitrogen. The dried residue was reconstituted with 50 ml of Milli-Q water (Millipore). A 20-µl aliquot of the solution was injected into the high-performance liquid chromatography-ultraviolet (HPLC-UV) detection system.

To collect the bile for a prolonged period, the bile was collected by a duct-cannulated rat model under anesthesia. Exteriorization of a PE10 tube was prepared before bile duct catheterization. A PE10 cannula was fixed to the bile duct (two positions at near liver and duodenum) by a surgical knot, and the wound was closed by sutures after duct cannulation was completed. The bile of the rat flowed into the circulation system when the pipes of the bile ducts were connected. 5-FU was administered *via* a PE50 cannula into the femoral vein, and blood was collected through a PE50 cannula from the jugular vein.

### Serum Cytokine Analysis

The plasma levels of cytokines [transforming growth factor beta 1 (TGF-β1), tumor necrosis factor alpha (TNF-α)] and matrix metalloproteinase-8 (MMP-8) obtained from the mouse blood samples were analyzed using enzyme-linked immunosorbent assay (ELISA) (R&D Systems) according to the manufacturer’s instructions.

## Data Analysis

The pharmacokinetic parameters were determined by calculating each individual set of data with a non-compartmental model using WinNonlin Standard Edition Version 1.1 software (Scientific Consulting Inc., Apex, NC). The pharmacokinetic parameters calculated were the initial drug concentration of 5-FU (*C*_0_), the maximum concentration and time of 5- FDHU (*C*_max_, t_max_), the area under the concentration versus time curve (AUC), the clearance (CL), the elimination half-life (*t*_1/2_), the volume of distribution at steady state (Vss) and the mean residence time (MRT). The statistical analyses were performed using analysis of variance in the SPSS 18.0 program (SPSS Inc., Chicago, USA) and SigmaPlot 10.0 software. All data are expressed as the mean ± standard deviation (S.D.). One-way ANOVA was used for the comparison between groups, and statistically significant differences were defined as ^*^ *P* < 0.05 or ^**^ *P* < 0.01.

## Results

### Chromatographic Analysis and Method Validation

**Figure 2:**
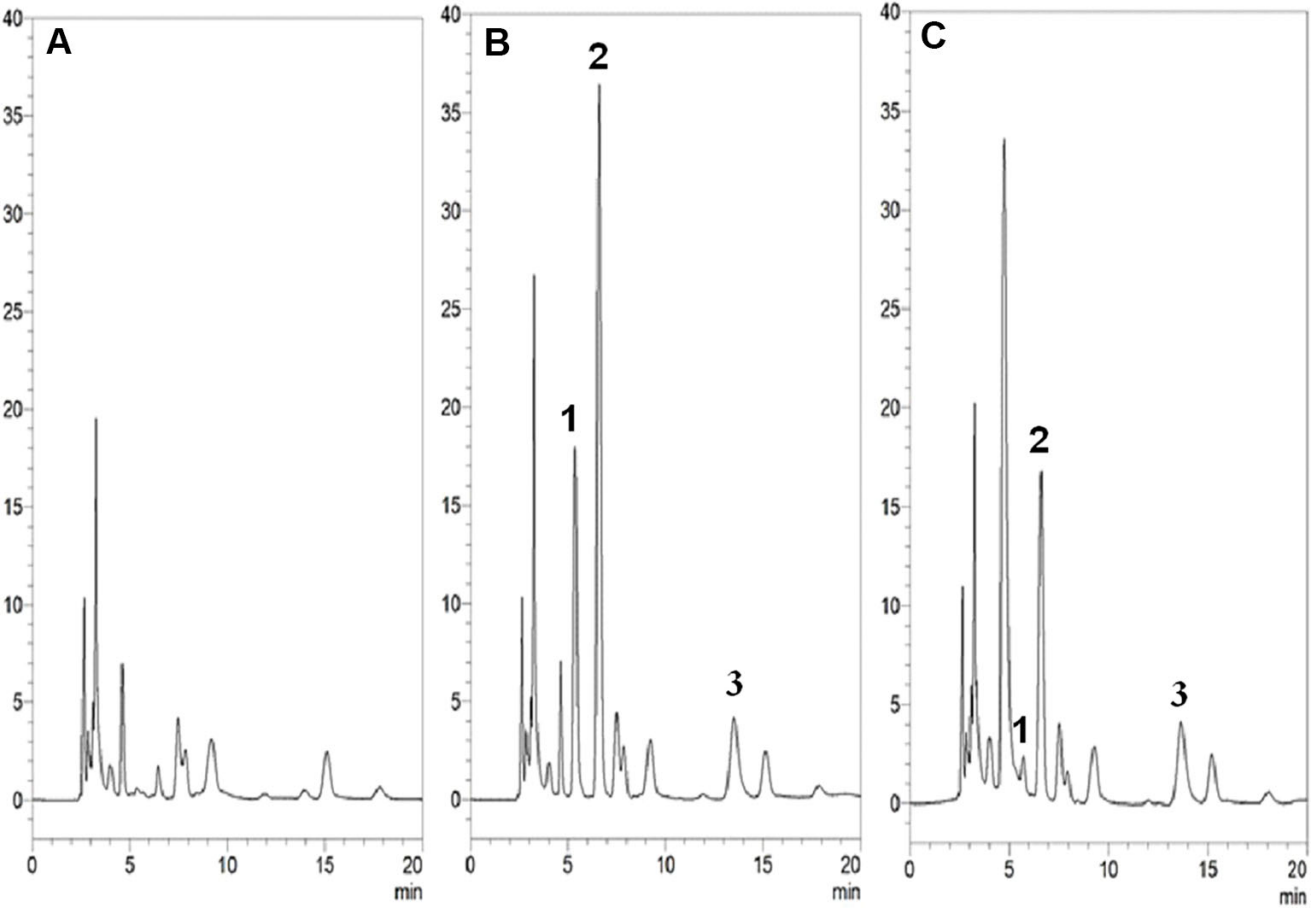
HPLC-UV chromatograms of **(A)** blank plasma samples; **(B)** blank plasma samples spiked with 5-FU, 5-FDHU (10 µg/ml) and internal standard (10 µg/ml); **(C)** blood sample containing 5-FU (7.9 µg/ml) and 5-FDHU (1.1 µg/ml) collected at 60 *min* after 5-FU (100 mg/kg, i.v.) administration alone. Peak 1: 5-FDHU and retention time of 5-FDHU was 5.3 *min*. Peak 2: 5-FU and retention time of 5-FU was 6.2 *min*. Peak 3: amoxicillin and retention time of amoxicillin was 12.4 *min*.

The mobile phase of 5% methanol and 95% 10 mM KH_2_PO_4_ (v/v) (pH 4.7) with a C18 column produced acceptable separation of 5-FU and 5-FDHU in the experiment. The respective retention times of 5-FU and 5-FDHU were 6.8 and 5.8 *min*, with good separation and no endogenous interference in the rat plasma samples, with good selectivity (**Figures 2A–C**). In the current study, the LODs of 5-FU and 5-FDHU in the plasma and in the bile were both 0.05 μg/ml. Good linearity of the calibration curves (*r* ^2^ > 0.999) over the range of 0.1−50 μg/ml was noted (**Table 1**). The absolute recoveries of 5-FU and 5-FDHU in plasma ranged from 102.9 to 103.2% and 99.3 to 105.4%, respectively. Additionally, the absolute recoveries of 5-FU and 5-FDHU in bile ranged from 107.4% to 113.7% and 103.9% to 108.0%, respectively (**Table 2**). The intra- and inter-day precision and accuracy values of 5-FU and 5-FDHU in the plasma and in the bile were within 15% (**Tables 3** and **4**).

**Table 1:** Linear ranges, calibration curves, correlation coefficients (*r* ^2^), and detection limits of 5-FU and FUH_2_ using HPLC-UV in the plasma and bile.

Compounds	Linear range (µg/ml)	Calibration curve	*r* ^2^	LLOQ (µg/ml)	LOD (µg/ml)
Plasma					
5-FU	0.1-50	y = 0.3263x + 0.0004	1	0.1	0.05
5-FDHU	0.1-50	y = 0.1907x + 0.0047	1	0.1	0.05
Bile					
5-FU	0.1-50	y = 0.3693x - 0.0011	1	0.1	0.05
5-FDHU	0.1-50	y = 0.2003x - 0.0009	1	0.1	0.05

LLOQ, lower limit of quantification was determined at a signal-to-noise ratio (S/N) of 10; LOD, limit of detection was determined at a signal-to-noise ratio (S/N) of 3.

**Table 2:** Extraction recoveries of 5-FU, 5-FDHU and amoxicillin (I.S.) from rat plasma and bile.

Con. (µg/ml)	Spiked in the mobile phase (Set 1)	Spiked before extraction (Set 2)		Recovery (%)	
		Plasma	Bile	Plasma	Bile
5-FU					
0.5	16137 ± 74.61	16611 ± 353.8	17330 ± 1335	102.9 ± 0.020	107.4 ± 0.083
5	156962 ± 7203	161181 ± 1401	17748 ± 20860	102.8 ± 0.042	113.7 ± 0.019
50	1598225 ± 37459	164982 ± 28128	1761897 ± 109320	103.2 ± 0.023	110.3 ± 0.065
5-FDHU					
0.5	8979 ± 337.7	9777 ± 153.0	9473 ± 437.6	109.0 ± 0.029	105.6 ± 0.052
5	88523 ± 5376	97537 ± 1124	5299 ± 3166	110.4 ± 0.057	108.0 ± 0.097
50	892893 ± 58184	963719 ± 10026	926613 ± 43317	108.2 ± 0.062	103.9 ± 0.028
I.S.					
10	98247 ± 1942	98929 ± 815.3	103691 ± 3631	100.7 ± 0.028	100.9 ± 0.071

Data are expressed as the means ± S.D. (n = 3). I.S.: amoxicillin; the recovery (%) = (the peak area of Set 2/the peak area of Set 1)*100.

**Table 3:** Inter-day and intra-day assay precision (% RSD) and accuracy (% Bias) values for the HPLC-UV method for the determination of 5-FU and 5-FDHU in rat plasma.

Nominal Con. (µg/ml)	Intra-day (n = 6)			Inter-day (n = 6)		
	Observed con. (µg/ml)	Accuracy Bias (%)	Precision RSD (%)	Observed con. (µg/ml)	Accuracy Bias (%)	Precision RSD (%)
5-FU						
0.1	0.095 ± 0.01	-4.587	2.612	0.090 ± 0.01	-10.14	12.12
0.5	0.506 ± 0.02	1.284	3.700	0.475 ± 0.04	-5.079	9.031
1	0.959 ± 0.06	-4.1485	6.638	0.921 ± 0.09	-7.915	9.968
5	4.999 ± 0.18	-0.016	3.524	4.912 ± 0.31	-1.754	6.319
10	10.07 ± 0.21	0.731	2.036	10.45 ± 0.60	4.533	5.732
50	50.04 ± 0.05	0.073	0.093	50.55 ± 1.32	1.106	2.604
5-FDHU						
0.1	0.112 ± 0.01	11.61	11.04	0.105 ± 0.01	4.740	12.12
0.5	0.528 ± 0.03	5.561	5.513	0.507 ± 0.03	1.430	5.027
1	1.043 ± 0.11	4.253	10.16	1.055 ± 0.10	5.543	9.004
5	5.082 ± 0.15	1.638	2.904	5.089 ± 0.16	1.784	3.116
10	9.819 ± 0.20	-1.814	2.021	9.975 ± 0.31	-0.248	3.085
50	50.05 ± 0.16	0.104	0.323	50.17 ± 0.54	0.343	1.086

Data are expressed as the means ± S.D. (n = 6) Precision (%RSD) = S.D./C_obs_ *100. Accuracy (%Bias) = (C_obs_-C_nom_)/C_nom_ *100.

**Table 4:** Inter-day and intra-day assay precision (% RSD) and accuracy (% Bias) values for the HPLC-UV method for the determination of 5-FU and 5-FDHU in rat bile.

Nominal Con. (µg/ml)	Intra-day (n = 6)			Inter-day (n = 6)		
	Observed con. (µg/ml)	Accuracy Bias (%)	Precision RSD (%)	Observed con. (µg/ml)	Accuracy Bias (%)	Precision RSD (%)
5-FU						
0.1	0.100 ± 0.01	-0.403	2.298	0.098 ± 0.01	-2.166	6.898
0.5	0.499 ± 0.01	-0.262	2.300	0.513 ± 0.03	2.601	6.343
1	1.014 ± 0.02	1.357	2.337	1.041 ± 0.06	4.055	6.081
5	4.986 ± 0.08	-0.272	1.578	4.866 ± 0.22	-2.670	4.617
10	10.02 ± 0.07	0.184	0.686	10.09 ± 0.33	0.903	3.236
50	50.05 ± 0.05	0.100	0.091	50.01 ± 0.06	0.014	0.117
5-FDHU						
0.1	0.098 ± 0.01	-1.923	9.729	0.095 ± 0.01	-4.625	8.980
0.5	0.506 ± 0.01	1.225	2.305	0.513 ± 0.02	2.563	4.851
1	0.994 ± 0.03	-0.638	2.657	1.053 ± 0.10	5.305	9.268
5	4.945 ± 0.16	-1.092	3.264	4.958 ± 0.44	-0.850	8.871
10	10.10 ± 0.21	0.988	2.061	10.00 ± 0.54	0.021	5.413
50	50.11 ± 0.63	0.225	0.126	50.09 ± 0.13	0.172	0.269

Data are expressed as the means ± S.D. (n = 6) Precision (%RSD) = S.D./C_obs_ *100. Accuracy (%Bias) = (C_obs_-C_nom_)/C_nom_ *100.

### RT-PK Pharmacokinetic Interaction Study

**Figure 3:**
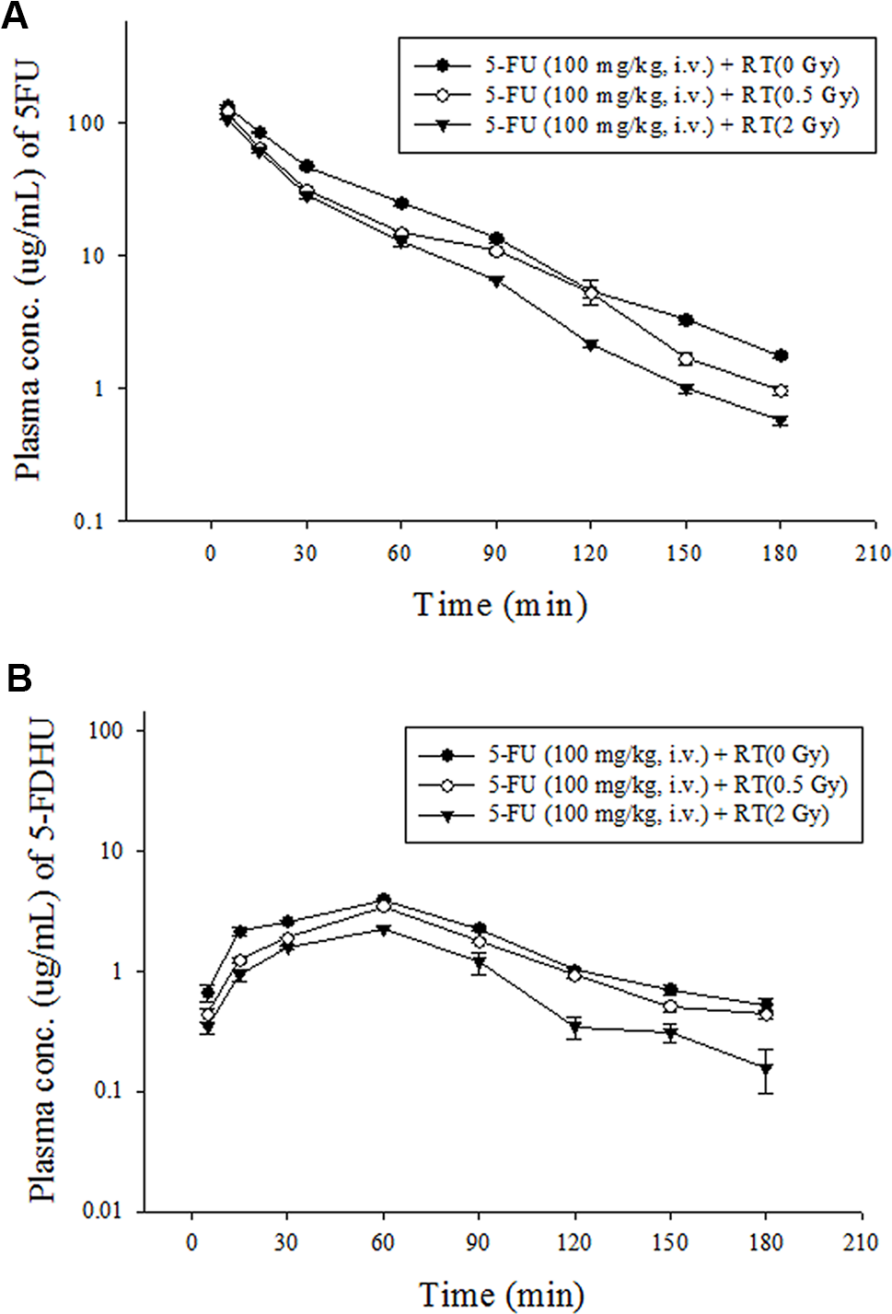
**(A)** Mean plasma concentration-time curve of 5-FU (•) in rat blood after 5-FU administration (100 mg/kg, iv) alone, 5-FU (100 mg/kg, i.v.) + RT (0.5 Gy) (○) and 5-FU (100 mg/kg, i.v.) + RT (2 Gy) (▼). **(B)** Mean plasma concentration-time curve of 5-FDHU (•) in rat blood after 5-FU administration (100 mg/kg, iv) alone, 5-FU (100 mg/kg, i.v.) + RT (0.5 Gy) (○) and 5-FU (100 mg/kg, i.v.) + RT(2 Gy) (▼). (n = 6).

The radiation at 2 Gy was the daily treatment dose for a human, and 0.5 Gy simulated the off-target dose in clinical practice. Compared with the sham RT group, as in previous reports (Hsieh et al., 2011; Hsieh et al., 2015), the current study also confirmed that local pelvic irradiation decreased the AUC_plasma_ of 5-FU by 23.7% at 0.5 Gy (P < 0.001) and 35.3% at 2 Gy (P < 0.001). Intriguingly, the AUC_plasma_ of 5-FDHU was also reduced by 21.4% at 0.5 Gy (P < 0.001) and 51.5% at 2 Gy (P < 0.001). Additionally, pelvic irradiation significantly increased the clearance values (CLs) and the volume of distribution at steady state (Vss) of 5-FU by 30.6% and 16.7% at 0.5 Gy and 50.1% and 17.7% at 2 Gy, respectively. Meanwhile, the CLs of 5-FDHU were increased by 27.2% at 0.5 Gy and 106% at 2 Gy. The MRTs of 5-FU and 5-FDHU in the plasma were decreased by 23.7% at 0.5 Gy and 18.2% at 2 Gy, respectively (**Figures 3A, B**; **Table 5**).

**Table 5:** Pharmacokinetic parameters of 5-FU (100 mg/kg, i.v.) and 5-FDHU from rat plasma.

Parameter	Unit	Control		Whole pelvic RT 0.5 Gy		Whole pelvic RT 2 Gy	
		5-FU	5-FDHU	5-FU	5-FDHU	5-FU	5-FDHU
AUC	min μg/ml	5114 ± 109	372.7 ± 8.43	3904 ± 84.8**	292.6 ± 3**	3307 ± 68.5**	181.4 ± 10**
C_0_		169.3 ± 8.52	–	168.3 ± 11.6	–	142.1 ± 3,1**	–
C_max_	μg/ml	–	3.94 ± 0.02	–	3.45 ± 0.1**	–	2.27 ± 0.1**
T_max_	min	–	60	–	60	–	60
t_½_	min	34 ± 5	48 ± 9	27 ± 2	38 ± 1*	28 ± 4**	31 ± 2**
Cl	ml/min/kg	19.56 ± 0.41	268.4 ± 6.01	25.62 ± 0.5**	341.7± 3**	30.24 ± 0.61**	552.8 ± 33**
Vss	ml/kg	742.3 ± 26.1	–	866.1 ± 37**	–	873.3 ± 19**	–
MRT	min	38 ± 1	88 ± 5	34 ± 1	86 ± 1	29 ± 0.45**	72 ± 3**

AUC, area under the plasma concentration vs. time curve; t1/2, terminal elimination phase half-life; Cmax, maximum observed plasma concentration; MRT, mean residence time; CL, total plasma clearance; Vss, volume of distribution at steady state. *significantly different from the without RT group at p < 0.05; **significantly different from without RT group at p < 0.01.

**Figure 4:**
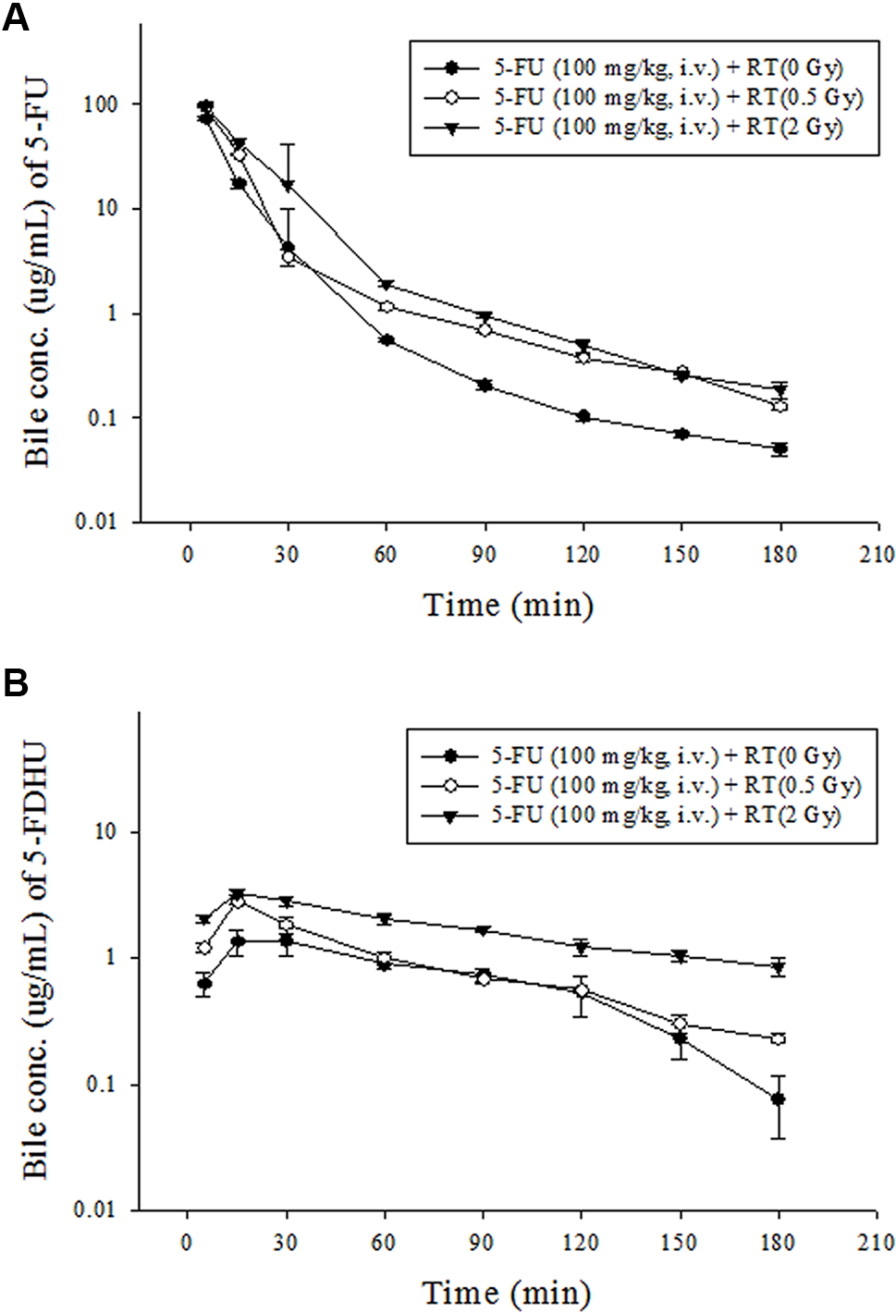
**(A)** Mean bile concentration-time curves of 5-FDHU (•) in rat bile after 5-FU administration (100 mg/kg, iv) alone, 5-FU (100 mg/kg, i.v.) + RT (0.5 Gy) (○) and 5-FU (100 mg/kg, i.v.) + RT(2 Gy) (▼). **(B)** Mean bile concentration-time curves of 5-FDHU (•) in rat bile after 5-FU administration (100 mg/kg, iv) alone, 5-FU (100 mg/kg, i.v.) + RT (0.5 Gy) (○) and 5-FU (100 mg/kg, i.v.) + RT(2 Gy) (▼). (n = 6).

In contrast, the AUCs of 5-FU in the bile were increased by 36.7% at 0.5 Gy (P < 0.001) and 68.6% at 2 Gy (P = 0.005). Meanwhile, the AUCs of 5-FDHU in the bile were increased by 40.3% at 0.5 Gy (P < 0.001) and 248.1% at 2 Gy (P < 0.001). Additionally, pelvic irradiation significantly increased the respective MRTs of 5-FU in the bile by 60.0% at 0.5 Gy and 125.0% at 2 Gy. Meanwhile, the clearance of 5-FU in the bile was increased by 31.8% at 0.5 Gy and 11.2% at 2 Gy. However, the clearances of 5-FDHU in the bile were decreased by 29.1% at 0.5 Gy and 71.0% at 2 Gy (**Figures 4A, B**; **Table 6**).

**Table 6:** Pharmacokinetic parameters of 5-FU (100 mg/kg, i.v.) and 5-FDHU from rat bile.

Parameter	Unit	Control		Whole pelvic RT 0.5 Gy		Whole pelvic RT 2 Gy	
		5-FU	5-FDHU	5-FU	5-FDHU	5-FU	5-FDHU
AUC	min μg/ml	1263 ± 121	128.5 ± 36.2	1727 ± 120*	181.4 ± 6.25	2129 ± 591**	449.4 ± 65**
C_0_		147.2 ± 3.39	–	159.3 ± 5.81	–	145.6 ± 10.23	–
C_max_	μg/ml	–	1.62 ± 0.11	–	2.79 ± 0.2**	–	3.28 ± 0.2**
T_max_	min	–	23 ± 8	–	15	–	15
t_½_	min	59 ± 10	27 ± 10	38 ± 1**	48 ± 6	35 ± 3**	107 ± 34**
Cl	ml/min/kg	43.78 ± 12.7	778.9 ± 22.1	58.13 ± 3.8**	551.9 ± 18**	49.15 ± 9.4**	226 ± 65.2**
Vss	ml/kg	768.6 ± 56.6	–	794.0 ± 3.85	–	750.8 ± 70.6	–
MRT	min	10 ± 2	67 ± 8	14 ± 4	74 ± 5	16 ± 3**	151 ± 43**

AUC, area under the plasma concentration vs. time curve; t1/2, terminal elimination phase half-life; Cmax, maximum observed plasma concentration; MRT, mean residence time; CL, total plasma clearance; Vss, volume of distribution at steady state. *significantly different from the without RT group at p < 0.05; ** significantly different from the without RT group at p < 0.01.

### Cytokine Response During RT-PK of 5-FU

Compared with the sham group, there were no significant differences in TGF-β1 and TNF-α between the RT 0.5 Gy followed by 5-FU and RT 2 Gy followed by 5-FU groups. MMP-8 expression increased by 27.7% in the RT 0.5 Gy followed by 5-FU (7319 ± 3473) group. However, MMP-8 expression increased by 100% in the RT 2 Gy followed by 5-FU (11494 ± 2606) group when compared with the sham group (5733 ± 2656, p=0.005).

### White Blood Cell, Hemoglobin, Platelet, Creatine, and Hepatic Function After RT 0.5 Gy or 2 Gy With 5-FU Treatment

The serum concentrations of white blood cell (K/μl), hemoglobin (g/dL), platelet (K/µl), creatine (mg/dl) and alanine aminotransferase (U/L) levels between the 5-FU-treated versus (vs.) 0.5 Gy followed by 5-FU-treated vs. 2 Gy followed by 5-FU-treated were 6.42 ± 0.26 vs. 6.28 ± 0.15 vs. 6.09 ± 2.71, 8.02 ± 1.20 vs. 8.80 ± 1.56 vs. 8.55 ± 2.54, 401.8 ± 127.4 vs. 427.8 ± 110.9 vs. 367.0 ± 136.8, 0.20 ± 0.17 vs. 0.55 ± 0.26 vs. 0.10 ± 0 and 64.4 ± 22.9 vs. 75.0 ± 32.1 vs. 61.8 ± 28.4, respectively. There were no significant differences between the 5-FU-treated versus (vs.) 0.5 Gy followed by 5-FU-treated vs. 2 Gy followed by 5-FU-treated groups.

## Discussion

5-FU is commonly used to enhance RT effects (Wolmark et al., 2000; Bosset et al., 2006). The RT-PK phenomenon of 5-FU is a phenomenon in which systemic 5-FU could be modulated by local irradiation with a change in the AUC of 5-FU in plasma (Hsieh et al., 2011; Hsieh et al., 2015). However, whether the metabolism of 5-FU is modulated by RT is still unclear (Hsieh et al., 2015). Here, local pelvic irradiation reduced the AUC_plasma_ values of 5-FU by 24% and 35% at 0.5 Gy and 2 Gy, respectively. The AUC_plasma_ values of 5-FDHU, the metabolite of 5-FU, also declined by 21% at 0.5 Gy and 52% at 2 Gy. Additionally, the AUC_bile_ values of 5-FU increased by 37% and 69% at 0.5 Gy and 2 Gy, respectively. Meanwhile, the AUC_bile_ values of 5-FDHU increased by 40% at 0.5 Gy and 248% at 2 Gy. The current study reconfirmed the RT-PK phenomenon of 5-FU and suggested that the metabolite of 5-FU, 5FDHU, can be modulated by RT, with similar trends in the two compounds.

There are correlations between plasma levels of 5-FU and treatment outcomes (Milano et al., 1994; Gamelin et al., 1998). Higher AUC values of 5-FU are associated with impressive survival and response rates (Milano et al., 1994; Gamelin et al., 1998). However, 31% to 34% of treated patients have dose-limiting toxicities (Meta-Analysis Group In et al., 1998). Concomitant administration of 5-FU and RT increases the rate of observed grade 3 or higher acute mucositis (Strigari et al., 2016). Bazan et al. (2013) reported that acute hematologic toxicity in patients treated with pelvic RT concomitant with 5-FU was 8% higher than in those undergoing RT alone. Prolonged tumor retention of 5-FU and the enhanced cytotoxicity followed by RT was reported by Blackstock et al. (1996).

Actually, as the ratio of AUC of 5-FU/5-FDHU increased, the risk of adverse events in cancer patients also increased (Di Paolo et al., 2001). The current study showed that the 5-FU/5-FDHU AUC ratios in the sham, 0.5 Gy and 2 Gy group were 13, 13 and 18, respectively. Additionally, pelvic irradiation significantly increased the respective Vss of 5-FU by 16.7% at 0.5 Gy and 17.7% at 2 Gy. According to the above data (Blackstock et al., 1996; Di Paolo et al., 2001), the current RT-PK phenomenon supported that RT plus 5-FU could cause more adverse events than 5-FU alone by increasing the ratio of AUC of 5-FU/5-FDHU and the Vss of 5-FU. Additionally, the off-target dose also modulates the PK of 5-FU and contributes to the toxicity during CCRT.

Bocci et al. (2006) noted that there are significant correlations between the AUCs of 5-FU and 5-FDHU. Additionally, 5-FDHU plasma levels may correlate with DPD activity weakly but significantly (Bocci et al., 2006). Meanwhile, DPD activity is determined by the CL of 5-FU in plasma, and high-5-FU CL predicts lower toxicity and poor outcome for colorectal cancer patients (Gusella et al., 2009). The current study showed that pelvic irradiation, whether 0.5 Gy or 2 Gy, increased the CL of 5-FU. Meanwhile, the AUC_plasma_ of 5-FDHU declined by both irradiation doses. Additionally, the CL of 5-FDHU also increased by irradiation. In contrast to the values in the plasma, the AUCs of 5-FU and 5-FDHU in the bile were increased by irradiation. This suggests that the activity of DPD may be decreased by irradiation and may be dose-dependent. Irradiation may reduce the toxicity of 5-FU by decreasing the AUC_plasma_ of 5-FU and increasing the bile excretion of 5-FU. However, the decreased AUC of 5-FU may cause poor clinical outcomes (Milano et al., 1994; Gamelin et al., 1998; Gusella et al., 2009), suggesting that RT followed by 5-FU is not an ideal model in clinical practice.

There were no differences in the levels of TGF-β1 and TNF-α between the RT and sham groups. However, the expression of MMP-8 increased by 100% at 2 Gy when compared with the sham group. Irradiation causes bystander signaling or abscopal effects through interleukins, cytokines, reactive oxygen species, TGF-β1 and TNF-α (Chen et al., 2017). MMPs degrade extracellular matrix proteins. MMP activity can be upregulated by nitric oxide (NO)-mediated S-nitrosylation (Gu et al., 2002). MMP-8 is an proinflammatory mediator and mediates inflammatory processes (Lee et al., 2014). MMP-8 acts directly on the proinflammatory cytokine TNF-α, leading to cytokine production and inflammatory responses (Sriram and O’callaghan, 2007; Bahia and Silakari, 2010); MMP-8 is upregulated during hepatic ischemia and reperfusion injury (Cursio et al., 2002). A previous report suggested that MMP-8 may play a role in the RT-PK phenomenon (Hsieh et al., 2011). Interestingly, the current study confirmed the upregulated expression of MMP-8 in the plasma after local irradiation. However, there is very little known about the specific functions of MMP-8 in the RT–PK phenomenon.

Low-dose RT in clinical practice has become more popular with advanced radiotherapy techniques. However, off-target RT may produce unexpected or unwanted biological effects (Shueng et al., 2009; Darby et al., 2013). For example, the increased scattering of low-dose irradiation by esophageal stent during treatment increases the risk of aortic pseudoaneurysm formation and the risk of perforating the esophagus (Hou et al., 2015; Lu et al., 2018). An additional 1 Gy of radiation increases the incidence of major coronary events by 7.4% in breast cancer patients receiving RT (Darby et al., 2013). However, the biological effects of low-dose RT are still unclear. Here, the current study confirmed that even low-dose irradiation can modulate the PK of anticancer drugs and their metabolites and can facilitate the excretion of drugs to the bile.

There were some limitations to this study. First, the current study was designed to examine the interaction between RT and PK of 5-FU and 5-FDHU but did not include the pharmacodynamics of 5-FU and 5-FDHU during RT. The current study confirmed there were no significant differences of toxicity effects between 5-FU-treated and RT plus 5-FU-treated groups. The current data was compatible with the previous report (Hsieh et al., 2011). These data also provided clues about systemic toxicology during the RT-PK phenomenon. Second, the delivery of RT in the current study was a single fraction. Further study to mimic clinical practice by using continued RT delivery to optimize the timing, duration, and dosing of 5-FU and 5-FDHU in the RT-PK phenomenon is warranted. Third, the possible mechanism was not included in the current study, although the current study noted that MMP-8 expression in the plasma was upregulated in the 5-FU and 5-FDHU following RT group. However, we confirmed that the systemic PK of 5-FU and 5-FDHU could be modulated by RT with sequential administration. Finally, the decreased AUC of 5-FU may cause poor clinical outcomes; nevertheless, it is not supported by the current experimental evidence. The current results just confirm that irradiation could modulate the pharmacokinetics of 5-FU and 5-FDHU. Further studies for detecting the optimal strategies, such as metronomic (Bocci and Kerbel, 2016), concurrent or sequential regimens for RT and 5-FU, are clearly still required in the future.

The current results show that both conventional and low-dose irradiation can modulate the pharmacokinetics of 5-FU and 5-FDHU. Additionally, RT plus 5-FU could cause more adverse events than 5-FU alone by increasing the AUC ratio of 5-FU/5-FDHU and the Vss of 5-FU. Irradiation may reduce the toxicity of 5-FU by decreasing the AUC of 5-FU in the plasma and increasing the bile excretion of 5-FU. However, the decreased AUC of 5-FU may cause poor clinical outcomes, suggesting that a sequential regimen of RT and 5-FU is not an ideal model in clinical practice. Hopefully, the current study sheds new light on these effects and increases our understanding of the effects of low dosage in the era of highly advanced RT.

## Data Availability Statement

All datasets generated for this study are included in the article.

## Ethics Statement

The animal study was reviewed and approved by The Institutional Animal Experimentation Committee of National Yang-Ming University, Taipei, Taiwan, and by the Institutional Animal Care and Use Committee (IACUC, approval number 106DN22).

## Author Contributions

All of the authors have read and approved the final manuscript. Study design and data interpretation: J-HL, T-HT, and Y-JC. Data collection: J-HL. Data analysis: J-HL. Manuscript writing: J-HL and C-HH. Manuscript revision: L-YW and H-YL. Acquisition of funding: T-HT, Y-JC, L-YW, and C-HH.

## Conflict of Interest

The authors declare that the research was conducted in the absence of any commercial or financial relationships that could be construed as a potential conflict of interest.
